# Expression of miR-320 and miR-204 in myocardial infarction and correlation with prognosis and degree of heart failure

**DOI:** 10.3389/fgene.2022.1094332

**Published:** 2023-01-11

**Authors:** Yuanyuan Yang, Qiongya Guo, Min Lu, Yansheng Huang, Yu Yang, Chuanyu Gao

**Affiliations:** ^1^ Department of Cardiology, People’s Hospital of Zhengzhou University, Zhengzhou, Henan, China; ^2^ Department of Cardiology, Henan Provincial People’s Hospital, Zhengzhou, Henan, China; ^3^ Department of Gastroenterology, People’s Hospital of Zhengzhou University, Zhengzhou, Henan, China; ^4^ Department of Gastroenterology, Henan Provincial People’s Hospital, Zhengzhou, Henan, China; ^5^ Department of Geriatrics, The Second Xiangya Hospital, Central South University, Changsha, Hunan, China; ^6^ Department of Cardiology, Fuwai Central China Cardiovascular Hospital, Zhengzhou, Henan, China

**Keywords:** myocardial infarction, miR-320 and miR-204, healthcare strategy, medical data, internet of things (IoMT) medical data

## Abstract

Myocardial infarction is a very dangerous cardiovascular disease with a high mortality rate under the modern developed medical technology. miRNA is a small molecule regulatory RNA discovered in recent years, which can play an important role in many cancers and other diseases. Medical data, machine learning and medical care strategies supporting the Internet of Things (IoMT) have certain applications in the treatment of myocardial infarction. However, the specific pathogenesis of myocardial infarction is still unclear. Therefore, this paper aimed to explore the expression of microRNA-320 and microRNA-204 in myocardial infarction and used the expression of microRNA-320 and microRNA-204 to predict the prognosis of patients with myocardial infarction. In order to discuss the expression of microRNA-320 and microRNA-204 in myocardial infarction in more detail. In this paper, 40 patients in the trial period were selected for clinical research, and 10 patients with normal cardiac function were selected in NHF group as control group. 10 patients with heart failure were selected as AMHF group. 10 patients with acute myocardial infarction were selected as AMNHF group. 10 patients with heart failure after old myocardial infarction were selected as OMHF group. AMHF group, AMNHF group and OMHF group were taken as the case group. This paper analyzed the difference of miR between different groups and determined that there were significant differences in the expression of miR-320 and miR-204 between different groups. Finally, the expression and prognosis of miR-320 and miR-204 in myocardial infarction were analyzed. The analysis results showed that the expression of microRNA-320 and microRNA-204 can inhibit the activity of myocardial cells. On the fifth day, the corresponding expression of microRNA-320 and microRNA-204 reduced the optical density of myocardial cells to 1.75 and 1.76, which was significantly lower than that on the first day. Moreover, excessive miR-320 expression and excessive miR-204 expression can increase the apoptosis rate of myocardial cells. The above results indicated that the high expression of microRNA-320 and microRNA-204 can be a bad prognostic factor in patients with myocardial infarction, showing that medical data, machine learning and medical care strategies supporting IoMT can play a role in the treatment of myocardial infarction. Therefore, it is urgent to understand the pathogenesis of heart failure after myocardial infarction and find new treatment schemes to improve the positive prognosis.

## 1 Introduction

In developed countries, heart failure after myocardial infarction is an important cause of hospitalization and death. With the development of medical technology, the survival rate of patients has also been improved and the life cycle of patients with heart failure has also been extended. However, the aging population in China has led to an increasing incidence of heart failure year by year. Heart failure is a major public health problem facing China in the 21st century. In the past 10 years, although the treatment strategy of heart failure has changed from simply short-term hemodynamics to the goal of inhibiting and delaying the development of cardiac remodeling, its 5-year mortality is still close to that of malignant tumors. Therefore, to seek new therapeutic targets and reduce the mortality of patients are the most important for heart failure after myocardial infarction.

Myocardial infarction is still one of the diseases with the highest mortality rate, and there are many researches on the treatment of myocardial infarction. Fan Xiaoming studied the role of interleukin-10 in reducing renal injury after myocardial infarction in diabetes. The experimental results suggested that interleukin-10 is expected to become a detection index of myocardial infarction in diabetes (Fan et al., 2022). Reed Grant W studied the treatment and nursing of acute myocardial infarction. Through the analysis of the follow-up treatment results of 100 patients with acute myocardial infarction, he suggested that acute myocardial infarction should be nursed with traditional Chinese medicine to alleviate the pain of patients (Reed et al., 2017). Qiao Xiaoying explored the role of renal cortex matrix metalloproteinase-9 in the clinical treatment of myocardial infarction and believed that renal cortex matrix metalloproteinase-9 is an important factor to promote the recovery of myocardial injury after acute myocardial infarction (Qiao et al., 2022). Although there are many studies on the etiology and treatment of myocardial infarction, the specific pathogenesis is not particularly clear.

For the specific pathogenesis of myocardial infarction, many scholars have discussed the expression level of related factors. Dai Wangde showed the specific mechanism of myocardial ischemia in rats through the rat model of myocardial infarction, and he found that some factors had obvious relationship with the area of myocardial infarction and cardiac blood return in rats (Dai et al., 2022). Goncalves Meire S found that nerol can cause acute myocardial infarction in rats and can reduce the acute myocardial infarction induced by isoproterenol in rats by studying the rat model (Goncalves et al., 2022). Li Jianwei’s research indicated that the lack of breast cancer activating factor can aggravate the inflammatory reaction in mice with acute myocardial infarction. He analyzed the expression of breast cancer activator before and after inflammation through the mouse myocardial infarction model (Li et al., 2022). It can be found that most scholars focus on common factors for the expression of myocardial infarction related factors, and there are not many studies on the relationship between MicroRNA (miRNA) and myocardial infarction.

This paper analyzed the expression of microRNA-320 and microRNA-204 in myocardial infarction, which was rarely studied by predecessors. In addition, microRNA-320 and microRNA-204 are frequently thought to have a stronger connection to cancer and have a role in the development of cancer. The expression of microRNA-320 and microRNA-204 in myocardial infarction, however, has not received much attention. Therefore, the research in this paper is of great significance for the treatment of future myocardial infarction and the subsequent research on miRNA.

## 2 miR-320, miR-204 and myocardial infarction

### 2.1 Medical data, machine learning and healthcare strategies supporting IoMT

In myocardial infarction, metabolic remodeling is regarded as a compensation of adaptive ability. However, as time goes on, it may become a harmful result, which can eventually destroy the energy metabolism of the heart and the systolic frequency of the heart. At the same time, the decrease of the fatty acid intake and oxidation level in patients with myocardial infarction and the increase of blood glucose concentration have an important relationship with metabolic remodeling. The myocardial diameter and the quality of myocardial cells in the late diastole are also related to metabolic remodeling. In myocardial cells of patients with myocardial infarction, the protein content is decreased, and main fatty acid transportation *β* Oxidase and the expression of miRNA are also decreased. In the myocardial biopsy, the blood glucose intake and metabolism of patients with dilated cardiomyopathy have been significantly improved. In addition, some studies have shown that improving the utilization of fatty acids and sugars is an effective means to treat heart failure after myocardial infarction. In recent years, scholars from China and other countries have discussed the changes of small molecule metabolite content, aiming to explore the pathogenesis of heart failure and find new therapeutic targets (Redfors et al., 2021).

Amino acids play a vital role in human vitality and biosynthesis. Some amino acid metabolism abnormalities, such as miRNA and branched chain amino acids, can activate the signal transduction pathway of rapamycin target protein, thus promoting myocardial remodeling and causing heart failure (Blankenberg et al., 2018). In this paper, the difference of plasma amino acid metabolite miRNA between patients with heart failure and those without heart failure after myocardial infarction was analyzed by liquid chromatography tandem mass spectrometry. The most different metabolites and the pathogenesis of heart failure were investigated by cell test. This paper analyzed the changes of amino acid metabolism in plasma of patients with heart failure after myocardial infarction were and found the related metabolic indexes, and the pathogenesis was discussed.

### 2.2 miR-320 and miR-204

miRNA is tiny molecules that contain about 22 different kinds of coding RNAs (Ferhat et al., 2022). MiRNA is not only involved in the regulation of cell metabolism, differentiation, growth and apoptosis, but also in the occurrence and development of many diseases, such as tumors. In recent years, it has been new discoveries about miRNA in terms of heart failure, arrhythmia, cardiomyocyte apoptosis, vascular regeneration, *etc.* At present, the function of miRNA in human heart disease has been studied from patients with heart failure. Heart failure after myocardial infarction is mainly due to heart pump failure, which can not provide sufficient blood supply for the heart. Heart failure and sudden cardiac death are related to pathological myocardial hypertrophy. Therefore, it is very meaningful to explore its molecular mechanism and find new targets. Cardiac hypertrophy is mainly due to the activation of signal transduction and transcriptional regulation in cells, which leads to the enlargement of myocardial cells and the increase of interstitial synthesis. From 2006 to now, the correlation between miRNA and myocardial hypertrophy and heart failure has received extensive attention (Palsson et al., 2020). Cell culture experiments have shown that different miRNAs play an important role in the pathological changes of cardiac hypertrophy.

The expression level of miRNA can predict the development of the disease and may be a new therapeutic target for cardiovascular disease. However, it is very difficult to detect the myocardial tissue abnormalities in patients at an early stage. Therefore, it is very important to find new miRNA research methods. In this paper, different stages of heart failure caused by myocardial infarction were selected through miRNA array method to detect and compare the miRNA array of each group. Several miRNAs with the most significant differences between groups were screened to find specific antibodies to heart failure at different stages. New markers of heart failure and new miRNA regulatory pathways have been explored.

### 2.3 Calculation method of heart failure degree

The degree of heart failure can be evaluated by myocardial energy expenditure (MEE) (Swain et al., 2020). MEE detection in clinical practice is simple, safe, cheap, stable and reusable. At present, there are many indicators to evaluate MEE, such as maximum myocardial oxygen consumption, left ventricular systolic wall pressure, systolic wall stress time integral, pressure volume work, maximum left ventricular pressure, average velocity of myocardial annular shortening, average velocity of myocardial annular contraction, pressure work index, pressure volume area, *etc.*

The left atrial end systolic stress is a non-invasive measurement method. In addition, the multispectral imaging technology can analyze the blood flow through the aorta and accurately measure the stroke volume and ejection time. Therefore, circumferential end-systolic wall stress (cESS) can be used to replace the left ventricular systolic tension, so that patients with myocardial infarction can be evaluated non-invasive. In addition, studies have proved that the left ventricular external work is in direct proportion to the average cardiac pressure and cardiac output (Cerisano et al., 2019).

The patient should lie on the left side to be examined by PHILIP 7500 color Doppler ultrasound. At the same time, ECG signals should be recorded. The standard profile recommended by the American Ultrasonic Association is adopted, and the S3 ultrasonic probe is adopted, with the probe frequency of 2.5 MHz. The main parameters required for the experiment are shown in Table 1.

**TABLE 1:** Main parameters required for the experiment.

Serial no	Abbreviation	Full name
1	LVEDV	Left ventricular end diastolic volume
2	LVESV	Left ventricular end systolic volume
3	IVS	Interventricular septal
4	PWTs	Posterior wall thickness at end-systole
5	LVIDd	Left ventricular internal diameter at end-diastole
6	LVIDs	Left ventricular internal diameter at end- systole
7	LVEF	Left ventricular ejection fraction
8	LVFS	Left ventricular fractional shortening
9	cESS	Circumferential end-systolic wall stress

LVEF, LVFS, cESS, and MEE, are finally calculated by the relevant indicators in Table 1. The calculation process is as follows.

LVEF:

$$LVEF=\frac{LVEDV-LVESV}{LVEDV}\times 100\%$$ (1)

LVFS:

$$LVFS=\frac{LVM}{BSA}$$ (2)

$$LVM=0.8\times 1.4\times \left[{\left(LVIDd+IVS+PWTs\right)}^{3}-LVIDd^{3}\right]+0.6$$ (3)

BSA=0.0061×height+0.0128×weight−0.1529 (4)

Among them, LVM represents the mass of left ventricle and BSA represents the body surface area.

cESS:

$$cESS=\frac{SBP\times {\left(LVIDs/2\right)}^{2}\times \left\{1+\frac{{\left(LVIDs/2+PWTs\right)}^{2}}{{\left(LVIDs/2+PWTs/2\right)}^{2}}\right\}}{{\left(LVIDs/2+PWTs\right)}^{2}-{\left(LVIDs/2\right)}^{2}}$$ (5)

SBP is the systolic pressure measured by Mercury cuff sphygmomanometer.

MEE:

$$MEE=cESS\times LVET\times LVSV\times \left(heart\;rate\right)\times 4.3\times 10^{-7}$$ (6)

LVSV is left ventricular stroke volume.

## 3 Experiment on expression of Mir-320 and Mir-204 in myocardial infarction

### 3.1 Test object

#### 3.1.1 Case group

The patients with myocardial infarction hospitalized in a hospital from October 2020 to October 2021 were selected as the research objects, and 40 patients were randomly selected as the observation objects in this experiment. According to the myocardial infarction’s progression, they were split into four groups. One group is the NHF group, which stands for the control group with normal myocardial function. Patients with heart failure, those with acute myocardial infarction, and those with heart failure following an older myocardial infarction make up the other three case groups. These groups are designated as AMHF group, AMNHF group, and OMHF group, respectively. All participants are aware of the experimental content and signed the informed consent form to agree and cooperate with the experiment.

#### 3.1.2 Inclusion criteria

Patients with myocardial infarction who meet the diagnostic criteria of coronary heart disease: there are typical chest tightness, chest pain and elevation of S and T segments of ECG (electrocardiogram), and coronary angiography result shows acute coronary artery occlusion. Acute myocardial infarction is evaluated according to the killip grade. The killip score of patients in the AMHF group is more than II, and the killip score of patients with AMNHF is I (Abbasloo et al., 2020).

Patients who have had an old myocardial infarction and are experiencing heart failure must fulfill two criteria. First, they should have a history of coronary heart disease and coronary intervention. Second, they should have a cardiac function score of Grade 3 or above as determined by the New York Heart Association (Engel and Rockson, 2020).

### 3.2 Experimental data collection

#### 3.2.1 Collection and numbering of plasma samples

The enrolled patients should keep stomach empty in the morning of the next day after entering the group. They were collected 3 mL of peripheral venous blood through an ethylene diamine tetraacetic acid anticoagulant catheter, and plasma was separated 2 h later. The collected plasma was transferred to a 1 mL standard non RNA enzyme centrifuge tube. In different centrifuge tubes, the plasma of five patients is 100 ul each, a total of 500 ul, which should be put in the refrigerator at - 80°C.

#### 3.2.2 miRNA labeling

**FIGURE 1:**
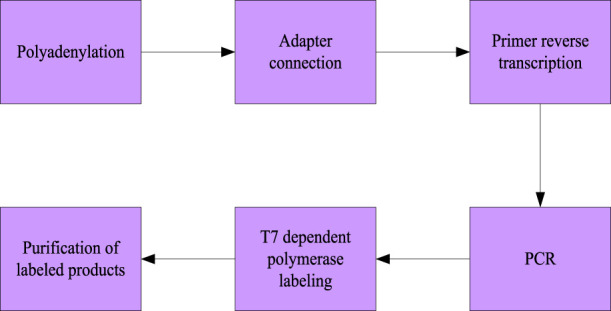
miRNA labeling steps.

The flow chart of miRNA labeling is shown in Figure 1. Specifically, it includes six steps: 1) 3′polyadenylation; 2) 5′adapter connection; 3) the oligo-dT primer containing a 3′adapter at the 5′end is used for reverse transcription; 4) PCR (polymerase chain reaction) detection is performed with primers containing promoters and complementary sequences of 3′and 5′adapters to the connected miRNA samples; 5) Polymerase dependent labeling, 6) purification of labeled products.

#### 3.2.3 Data analysis

The scanned image is extracted by GenePixPro6.0 software, and the average value is obtained by repeating 10 times. The miRNAs with a gene expression intensity of 50 or higher in all samples are medially normalized. The independent *t*-test is used to screen miRNAs. The Volcano Plot is obtained by taking Fold Change as the abscissa and the negative logarithm of P-log10 (*p*-value) as the ordinate. When the variation is more than 1.5 times, and *p* < .05, the gene expression difference between the two samples can be directly obtained.

All measurement data are expressed in terms of average standard deviation. When two measurement data conform to normal and variance, two independent t-tests are used respectively. If they do not conform, a modified *t*-test is used. S When multiple groups of measurement data conform to the positive and the square difference, the analysis of variance method is used; otherwise, the Welch test is used. PSS13.0 statistical software is used to analyze the data.

### 3.3 Differentiated miRNA

The general clinical data of different groups are shown in Table 2. It can be seen that the patients in the four groups have similar physical conditions. The BMI is about 22.5, and the *p*-value is 1.232. There is no difference. In the basic situation, such as age, height and sex, the *p*-value is greater than .05, indicating that there is no difference in the physical condition of each group of patients, and subsequent experiments can be carried out.

**TABLE 2:** General clinical data between different groups.

	NFH(10)	AMHF(10)	OMHF(10)	AMNHF(10)	*p*-value
Age	56.26	58.54	59.33	57.36	1.171
Height m)	1.62	1.64	1.65	1.65	1.986
Weight (kg)	55.34	55.87	57.54	52.78	.765
Male (proportion)	5 (50%)	4 (40%)	6 (60%)	5 (50%)	.875
BMI(kg/m^2^)	22.76	22.65	22.34	23.65	1.232

#### 3.3.1 Volcanic map for screening differential miRNA expression

**FIGURE 2:**
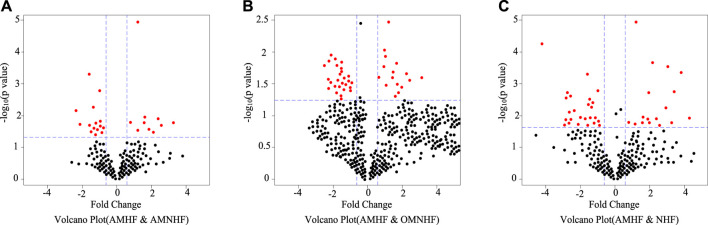
Volcanic map of differential expression miRNA. **(A)** Volcano Plot (AMHF & AMNHF) **(B)** Volcano Plot (AMHF & OMNHF) **(C)** Volcano Plot (AMHF & NHF).

Volcanic images are an effective means to intuitively display the differences between various samples. The index includes expression variation multiple and *p*-value, which can clearly reflect the relationship between different magnification (variation measurement) and statistical significance (taking into account the variation degree and variation degree). According to different data, they can be isolated from a group of special genes. In order to reveal miRNAs with significant differences in gene expression, volcanic maps of different groups were selected. The results are as shown in Figure 2. The blue dotted line is perpendicular to the coordinate axis, indicating a rise or decrease of 1.5 times, and the red dot indicates significant differential expression of miRNA. As shown in Figure 2A, the differentially expressed miRNAs in AMHF and AMNHF groups are more concentrated. As shown in Figures 2B,C, the differentially expressed miRNAs in AMHF and OMNHF groups are more dispersed.

#### 3.3.2 VDifferentially expressed miRNAs

MiRNA microarray analysis is a method to detect the difference of miRNA expression between patients with heart failure and normal control group by using peripheral venous blood plasma detection technology, so as to screen out differential miRNAs. The successful detection of differential miRNAs can find new heart failure markers, thus laying the foundation for new therapeutic goals.

As shown in Table 3, the five miRNAs with the largest difference in up regulation of expression between NHF group and AMNHF group are miR-409-3p, miR-26b-5p, miR-369-3p, miR-148b-3p and miRNA-329, respectively. The five miRNAs with the largest difference in down regulation of expression are miR-320, miR-204, miR-20b-3p, miR-767-5p and miR-299-5p, respectively.

**TABLE 3:** Differentially expressed miRNAs between NHF group and AMNHF group.

	Name	Fold change	*p*-value
Up regulation of expression	Hsa-mir -409-3p	6.653	.013
	Hsa-mir-26b-5p	5.765	.032
	Hsa-mir-369-3p	5.543	.035
	Hsa-mir-148b-3p	4.432	.041
	Hsa-mir-329	4.132	.047
Down regulation of expression	Hsa-mir-320	.765	.001
	Hsa-mir-204	.643	.005
	Hsa-mir- 20b-3p	.543	.014
	Hsa-mir-767-5p	.532	.018
	Hsa-mir -299-5p	.412	.023

As shown in Table 4, the five miRNAs with the largest difference in up-regulated expression between NHF group and OMHF group are miR-3941, miR-338-5p, miR-433, miR-19a-3p and miR-329 respectively. The five miRNAs with the largest down-regulation differences were miR-320, miR-204, miR-2110, miR-l247-5p and miR-296-5p, respectively.

**TABLE 4:** Differentially expressed miRNAs between NHF group and OMHF group.

	Name	Fold change	*p*-value
Up regulation of expression	Hsa-mir-3941	6.753	.015
	Hsa-mir-338-5p	5.755	.034
	Hsa-mir-433	4.546	.041
	Hsa-mir-19a-3p	4.432	.045
	Hsa-mir-329	4.165	.049
Down regulation of expression	Hsa-mir-320	.865	.003
	Hsa-mir-204	.748	.007
	Hsa-mir-2110	.663	.015
	Hsa-mir-l247-5p	.542	.019
	Hsa-mir-296-5p	.432	.025

As shown in Table 5, the five miRNAs with the largest difference in expression up regulation between NHF group and AMHF group are miR-3667, miR-4275, miR-647, miR-1284 and miR-1306-3p, respectively. The five miRNAs with the largest difference in expression down regulation are miR-320, miR-204, miR-205-5p, miR-423-5p and miR-21-3p, respectively.

**TABLE 5:** Differentially expressed miRNAs between NHF group and AMHF group.

	Name	Fold change	*p*-value
Up regulation of expression	Hsa-mir-3667-5p	8.753	.009
	Hsa-mir-4275	7.751	.013
	Hsa-mir-647	7.548	.019
	Hsa-mir-1284	6.433	.023
	Hsa-mir-1306-3p	6.165	.024
Down regulation of expression	Hsa-mir-320	.785	.007
	Hsa-mir-204	.758	.009
	Hsa-mir-205-5p	.675	.012
	Hsa-mir -423-5p	.641	.013
	Hsa-mir-21-3p	.572	.021

By comparing the difference miRNAs between the control group and different case groups, it can be found that there are significant differences in miR-320 and miR-204 between patients with normal myocardial function and patients with myocardial infarction, and this difference occurs in each course of disease. Therefore, it is meaningful to make targeted diagnosis and treatment of myocardial infarction through miR-320 and miR-204.

### 3.4 Expression of miRNA in myocardial infarction

#### 3.4.1 Construction and packaging of miRNA lentivirus

The selected gene information was miR-204-3p and miR-320C. The carrier information is GV254. The sequence of elements is Ubi-EGFP-MCS-IRES-Puromycin, and the cloning site is Nhe I.

The acquisition of target gene fragments is shown in Table 6.

**TABLE 6:** Target gene segment.

	Name	Seq
1	hsa-mir-204-p1	AGC​TGT​ACA​AGT​AAG​CCT​GAT​CAT​GTA​CCC​ATA​GG
2	hsa-mir-204-p1	GGG​AGA​GGG​GCT​TAG​CTT​ATG​GGA​CAG​TTA​TGG​GC
3	hsa-mir-320c	TAA​ACT​CGA​GGA​GGG​GGA​AAA​AAA​GAC​CTG​AT

The target gene is extracted from the plasmid containing the target gene by PCR technology, and the target vector is digested. After electrophoretic extraction, it is exchanged, and the final result is bacterial competent cells. The isolated clone is detected by PCR technology, and then the positive clone detected by PCR method is compared with it. If there is no error, the target gene can be obtained.

#### 3.4.2 Cell resuscitation and culture

The cells were separated from liquid nitrogen and quickly poured into 37°C water for rapid dissolution. It was then transferred to a centrifuge containing medium and centrifuged at room temperature for 5 min. The speed is 1,000 rpm. After centrifugation, the supernatant was removed and blown with fresh medium and then transferred to the culture bottle for culture in the incubator. 10% bovine serum protein and 1% penicillin streptomycin double antibody solution were used to culture in the medium at 37°C and saturated humidity. Cells with logarithmic growth cycles were used in the experiment.

In the case of high density, cell passage must be carried out. The suspension cells were discarded at a ratio of 1:2-1:4 and then cultured in a new complete medium. During passage, the old medium was discarded and washed with normal saline, and then .25% trypsin was added into the culture dish. When the cell volume increased, the complete culture medium was added. The cells were dried and then sent to a centrifuge, and then the supernatant was poured out, resuspended, and divided into a new medium at a ratio of 1:2-1:4.

#### 3.4.3 Cell cryopreservation

The cells were centrifuged at normal temperature for 5 min and then resuspended and transferred to a labeled refrigerated container. The container should be placed in a freezer at minus 80°C to transfer to liquid nitrogen the next day for long-term freezing.

#### 3.4.4 Cell proliferation test

Myocardial cells in logarithmic growth cycle were counted after suspension from body weight. Cells in the 24 well plate were seeded into the 24 well plate according to the density of 105 cells per well. Myocardial infarction differential miRNA and different concentrations of myocardial infarction drugs were added. At the same time, the blank culture medium and blank control group were set (only the same number of cells were added, without any other treatment) and placed in the culture dish for 48 h. The cells in the 24 well plate were transferred to the 96 well plate, 200 ul for each well, and four well plates in each group were incubated at 37°C for 3 h. The microplate reader was used to measure the optical density (OD) absorption value at the wavelength of 490 nm. Under stable transfection conditions, 200ul × 104 cells were placed on 96 well plates, and 20 ul nutrient solutions were added at different culture times. The OD value was determined after incubation at 37°C for 3 h.

#### 3.4.5 Detection of protein expression by western blot

##### 3.4.5.1 Extraction of whole cell protein

The cells of the three same treatment groups in the 6-well plate were gathered together for centrifugation and washed through the pre cooled PBS (phosphate buffered saline) to absorb the supernatant as much as possible. Next, the inhibitor of 1% phosphatase and protease was added, and the cells were subjected to ultrasonic wave for 5 s on the ultrasonic cell grinder after repeated blowing.

##### 3.4.5.2 Determination of sample protein concentration

18 ul PBS and two ul protein supernatant were added to the 96 well plate hole, and 200 ul premixed water-soluble compound working solution was added to fully mix and put in the 37°C incubator for 30 min. The protein concentration of each sample is calculated according to the standard curve formula and then multiplied by the corresponding dilution multiple to obtain the protein concentration.

##### 3.4.5.3 Protein electrophoresis

The bottom of 10% of the preform was torn to get a white adhesive strip, and then the rubber was added to the electrophoresis buffer in the electrophoresis tank. The liquid wass injected beyond the conductor. The comb was pulled out, and the comb hole was cleaned with electrophoresis buffer to remove foam and adhesive. According to the preset sampling sequence and feeding amount, the protein sample shall be placed in the comb hole marked with protein. At the beginning of electrophoresis, the constant voltage should be 80 V. When the protein is gradually separated, the voltage should be increased to 140 V until the electrophoresis is completed.

##### 3.4.5.4 Protein transfer

After electrophoresis, the glue, polyvinylidene fluoride membrane, filter paper and sponge pad were cleaned in the membrane rotation buffer solution to ensure that no bubbles are generated between each component. Next, the electrophoresis plate was placed in the groove of the rotary film buffer, ensuring that the adhesive faces black (cathode), and the polyvinylidene fluoride film faces white (anode), with a constant current of 350 mA. The membrane transfer time is usually 90–180 min, depending on the molecular weight of the target protein.

#### 3.4.6 Detection of target gene expression level

The myocardial cells were reverse transcribed into RNA and complex DNA. The PCR reaction system is shown in Table 7.

**TABLE 7:** PCR reaction system.

Serial no	Components	Volume (ul)
1	Upstream primer	.25
2	Downstream primer	.25
3	cDNA	1.00

#### 3.4.7 Expression of microRNA-320 and microRNA-204 in cardiomyocytes treated with demethylated drugs

**FIGURE 3:**
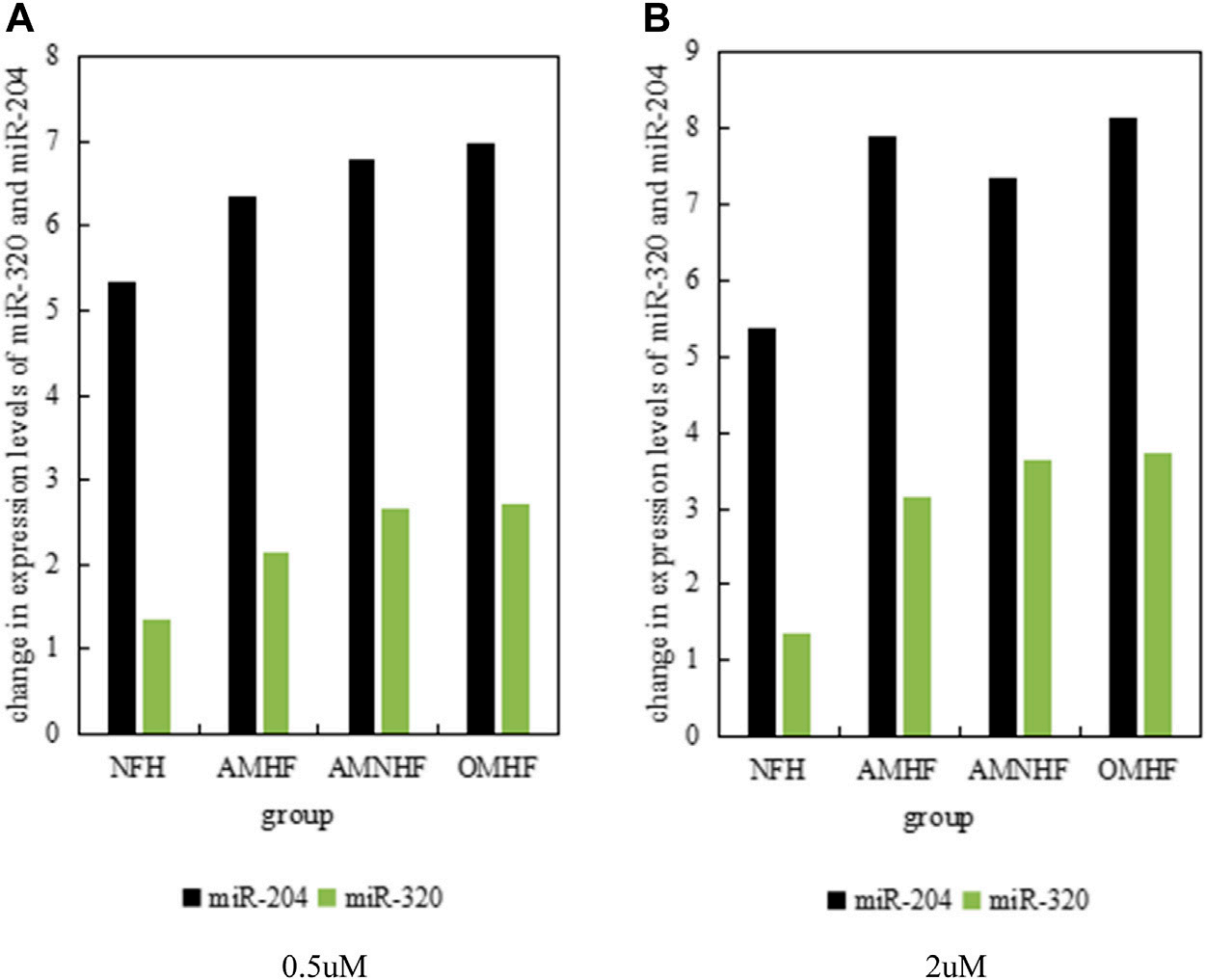
Expression of miRNA in each group at different concentrations. **(A)** 0.5uM **(B)** 2uM.

Demethylated drugs can play a certain role in the treatment of patients with myocardial infarction. It can be seen from the above text that compared with patients with normal myocardium, the expression levels of microRNA-320 and microRNA-204 in patients with myocardial infarction are significantly reduced. Therefore, .5 umol and two umol of demethylated drugs were used to treat myocardial cells, and the expression levels of microRNA-320 and microRNA-204 genes were studied. The results are as shown in Figure 3, and Figure 3A shows the situation that the drug is .5 umol; Figure 3B shows the situation where the drug is 2umol. It can be clearly found from Figure 3 that different concentrations of demethylated drugs have certain protective effects on myocardial cells and have certain effects on the expression levels of microRNA-320 and microRNA-204.

#### 3.4.8 Exogenous oligonucleotide transfection is highly utilized to express miRNA expression levels in myocardial cell lines

**FIGURE 4:**
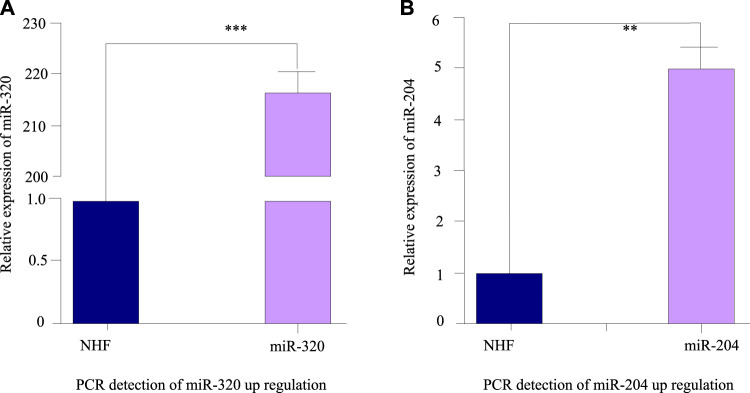
Fluorescence quantitative PCR detection results after miRNA transfection. **(A)** PCR detection of miR-320 up regulation. **(B)** PCR detection of miR-204 up regulation.

The results are shown in Figure 4. In subsequent experiments, oligonucleotides were used to transfect cardiomyocytes, and the expression of microRNA-320 and microRNA-204 in cardiomyocytes was detected by fluorescence quantitative PCR within 48 h. U6 was analyzed as an internal reference gene. As shown in Figure 4A, the level of miR-320 in myocardial cell lines transfected with oligonucleotides has been greatly improved. Compared with normal myocardial cells, the level of miR-320 has increased by about 215. As shown in Figure 4B, the miR-204 level of myocardial cell line transfected with oligonucleotides is 5 times higher than that of normal myocardial cells, which is significantly increased.

#### 3.4.9 Overexpression of miRNA inhibits the cell activity of myocardial cell lines

**FIGURE 5:**
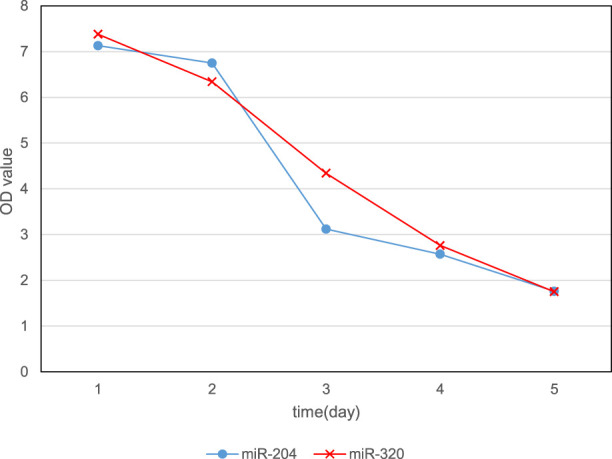
Change of OD value of cells in 5 days.

In order to analyze the specific effects of miRNA on cardiomyocytes, overexpression of miRNA was used to culture cardiomyocytes. The experiment was divided into 5 days, and the expression levels of microRNA-320 and microRNA-204 were gradually increased to observe the cell activity. The change of OD value for five consecutive days is shown in Figure 5. It can be found that with the increase of miR-320 and miR-204 expression levels over time, the OD values continued to decline, and the OD values on the fifth day were 1.75 and 1.76, respectively. This shows that the cell activity continues to decline, which means that the overexpression of miR-320 and miR-204 can reduce the activity of myocardial cells.

**FIGURE 6:**
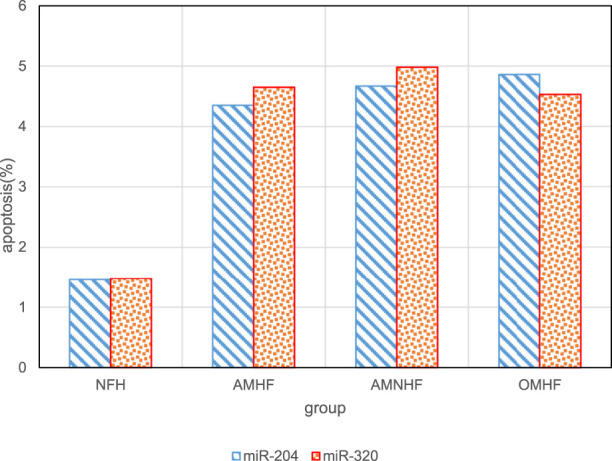
Relationship between overexpression of miR-320 and miR-204 and apoptosis in different groups.

In order to analyze the impact of overexpression of miR-320 and miR-204 on patients in different groups, the apoptosis rate of myocardial cells in the case of overexpression of miR-320 and miR-204 was tested respectively. The results are shown in Figure 6. The overexpression of miR-320 and miR-204 has a significant impact on the apoptosis rate of myocardial infarction patients.

#### 3.4.10 Comparison of myocardial function indexes between the two groups after treatment

At present, the function of miRNA in human heart disease has been studied from patients with heart failure. Heart failure is due to the pump failure of the heart, which can not provide sufficient blood supply for the heart and usually can lead to pathological cardiac hypertrophy and death.

Cardiac hypertrophy is an important manifestation of myocardial cells in various aspects such as blood pressure overload, endocrine disorder, myocardial injury, myocardial tissue and contractile protein gene mutation. Heart failure and sudden cardiac death are related to pathological myocardial hypertrophy (Kloner et al., 2020). Therefore, it is very meaningful to explore its molecular mechanism and find new targets. Cardiac hypertrophy is mainly due to the activation of signal transduction and transcriptional regulation in cells, leading to the enlargement of myocardial cells and the increase of interstitial synthesis.

**FIGURE 7:**
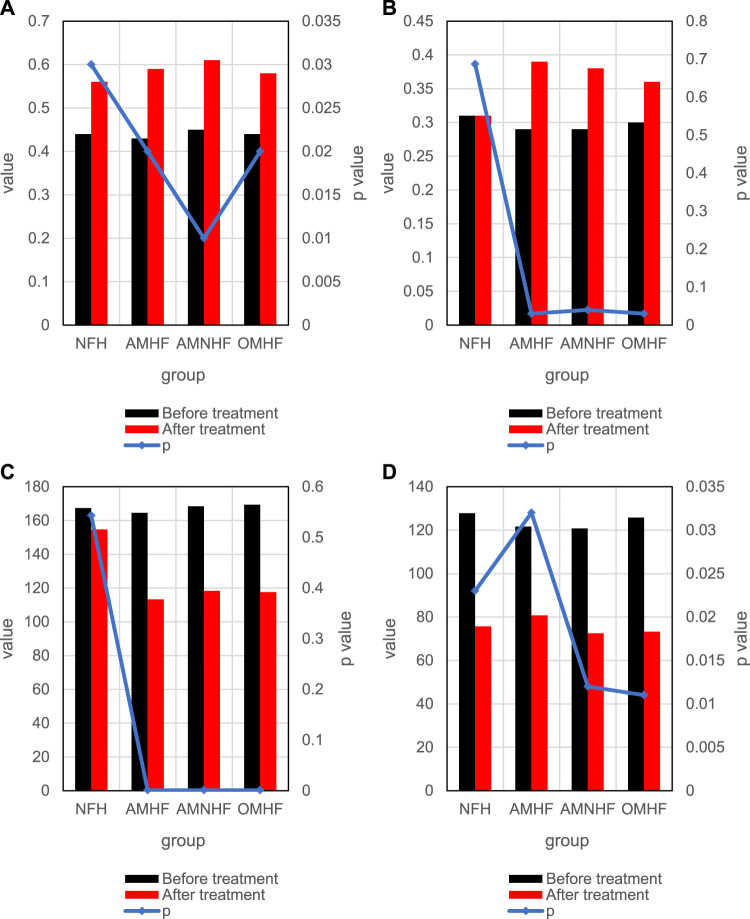
Comparison of differences between groups before and after treatment. **(A)** Changes of LVEF indicators in each group before and after treatment. **(B)** Changes of LVFS indicators in each group before and after treatment. **(C)** Changes of cESS indicators in each group before and after treatment. **(D)** Changes of MEE indicators in each group before and after treatment.

Figure 7 shows the changes of heart failure degree indicators in each group before and after treatment. Among them, Figure 7A shows the changes of LVEF indicators between groups before and after treatment, and Figure 7B shows the changes of LVFS indicators between groups before and after treatment; Figure 7C shows the changes of cESS indicators between groups before and after treatment, and Figure 7D shows the changes of MEE indicators between groups before and after treatment. As shown in Figure 7, the indicators LVFS and cESS are significantly different between the control group and the case group before and after treatment, which can be used as indicators to measure myocardial infarction.

The relationship between the expression level of target genes and the prognosis of patients with myocardial infarction.

**FIGURE 8:**
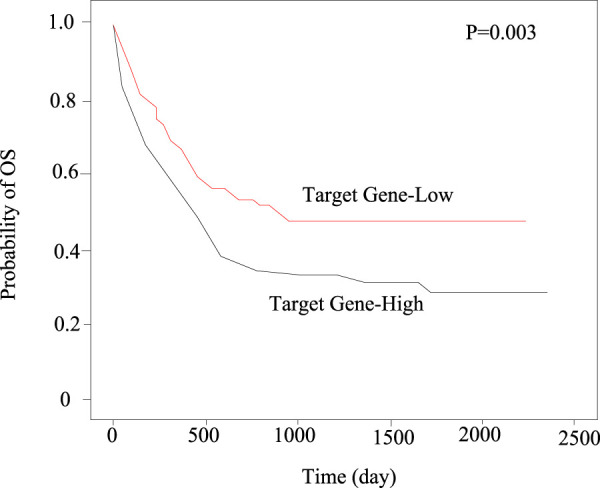
Survival graph.

In this paper, Kaplan-Meier method was used to compare the expression level of target genes in 30 patients with myocardial infarction. The results showed that the total survival period (OS) of the group with high expression of target genes is lower, as shown in Figure 8.

By analyzing the treatment response of patients with myocardial infarction in the high expression group and the low expression group of target gene, it can be found that the total survival period of patients with myocardial infarction in the high expression group is shorter. This shows that the role of high-level target genes in myocardial infarction is an important factor to affect the prognosis of myocardial infarction.

## 4 Conclusion

The human genome has more than 1,000 miRNAs, which regulate 1/3 of the human genes. MiR-1 and miR-133 are less expressed in genes, but MiR-126-3p, miR-125a, miR-125a, MiR-143, miR-320 and miR-204 are mainly expressed in tissue cell types. In other words, miRNA expression is different in different diseases. Therefore, the differential expression of miRNAs can be used to predict and diagnose diseases. In recent years, the abnormal expression of gene mRNA is closely related to cardiovascular diseases such as tumor, diabetes, heart failure, *etc.* In this paper, experiments have proved that miR-320 and miR-204 have differential expression in patients with myocardial infarction. Compared with people with normal myocardial cells, the expression levels of microRNA-320 and microRNA-204 in patients with myocardial infarction are significantly down regulated. The level of target gene expression corresponding to miR-320 and miR-204 has an important impact on the prognosis of myocardial infarction, and the expression of target gene transcripts is a negative factor for the prognosis of myocardial infarction. In this paper, there are still some deficiencies in the experiment, so in the follow-up research, more about medical data, machine learning and healthcare strategies supporting IoMT should be learned, hoping to better understand the pathogenesis of myocardial infarction.

## Data Availability

The original contributions presented in the study are included in the article/supplementary material, further inquiries can be directed to the corresponding author.
